# Atypical Reward-Driven Modulation of Mimicry-Related Neural Activity in Autism

**DOI:** 10.3389/fpsyt.2019.00327

**Published:** 2019-05-16

**Authors:** Janina Neufeld, Chun-Ting Hsu, Bhismadev Chakrabarti

**Affiliations:** ^1^Centre for Autism, School of Psychology and Clinical Language Sciences, University of Reading, Reading, United Kingdom; ^2^Karolinska Institutet Center of Neurodevelopmental Disorders (KIND), Centre for Psychiatry Research, Department of Women’s and Children’s Health, Karolinska Institutet and Stockholm Health Care Services, Stockholm County Council, CAP Research Centre, Stockholm, Sweden; ^3^Kokoro Research Center, Kyoto University, Kyoto, Japan

**Keywords:** autism, reward, mimicry, mirror system, fMRI, inferior frontal gyrus, social, conditioning

## Abstract

Autism spectrum disorder (ASD) is characterized by deficits in social functioning and difficulties in forming social bonds. According to the social motivation theory of ASD, people with ASD fail to attend social stimuli because they do not experience them as rewarding, resulting in deficits in social cognition. In neurotypical (NT) individuals, more rewarding faces have been shown to elicit greater spontaneous facial mimicry. This association between reward and mimicry is reduced in people with high autistic traits, suggesting that altered reward processing might explain the deficits in spontaneous facial mimicry observed in individuals with ASD. In a previous study, we observed that learned reward value of a face modulates mimicry-related neural response to it and that this modulation is reduced in people with high autistic traits. Using an identical evaluative conditioning paradigm where neutral faces were conditioned with high and low rewards, we tested the modulating effect of reward value on mimicry-related brain activity in a group of adults with and without ASD. We focused on the activity in a cluster within the inferior frontal gyrus (IFG) identified through an independent meta-analysis of 139 neuroimaging studies of mimicry, in response to passively viewing videos of the conditioned faces. The blood oxygen level dependent (BOLD) response contrast of high- vs. low-reward faces was reduced in participants with ASD compared to NT controls. The extent of reward-driven modulation was negatively correlated with autistic traits across the whole sample. Our results indicate that the mimicry-related brain response is less modulated by learned reward value in individuals with ASD when compared to NT controls. In previous studies, we found in a similar sample that being mimicked by faces was associated with less reward-related brain response in individuals ASD compared to an NT sample, suggesting that the link between reward and mimicry is affected in both directions in ASD. Together, this reduced bidirectional link between reward and mimicry can point to a potential mechanism underlying some of the social cognitive features of ASD.

## Introduction

Humans mimic each other automatically and unconsciously (1, 2). Mimicry leads to an increased feeling of closeness (1, 3), liking (4, 5) and more trust toward the mimicker (6, 7), as well as increased prosocial behavior (8–10). Hence, mimicry is believed to be crucial for forming social bonds (3, 11). Not only does mimicry increase liking but also vice versa, i.e., we mimic others more if we like them (12–14), suggesting a bidirectional link between mimicry and liking.

Mimicry of facial expressions and gestures can be overt or covert, i.e., characterized by small, invisible muscle contractions that can be measured with electromyography (EMG). The activation of motor-related brain regions while passively viewing others in action is often seen as a neural signature of mimicry (15). Using electroencephalography (EEG), the “mirroring” of others’ facial actions leads to an increase in mu suppression (16, 17). Functional neuroimaging techniques including positron emission tomography (PET) and functional magnetic resonance imaging (fMRI) have been used to record mimicry-related neural activity. A meta-analysis of functional neuroimaging studies on action observation and imitation experiments in humans, involving both hand movements and facial expressions, revealed a largely bilateral network of premotor, primary somatosensory, parietal, and temporo-occipital areas (15). Especially, the caudo-dorsal part of brodmann area (BA) within the inferior frontal gyrus (IFG) was consistently activated during both processes, suggesting this region as a core region for the overlap between action and action observation. This region is a key component of the putative “mirror system,” and is homologous to the macaque ventral premotor area F5, where mirror neurons were discovered originally (18–20). Mirror neuron response in F5 in monkeys is modulated by the reward value associated with the observed action (21). Similarly, reward value has been shown to modulate mimicry-related brain response in humans as indexed by mu suppression (22).

Lab-based experiments indicate that people who spontaneously mimic more tend to be better in recognizing emotions (23, 24) and that hindering people from mimicking spontaneously can impair their emotion recognition ability (25). Further, explicitly instructing people to mimic can enhance their ability to identify emotional facial expressions, especially among neurotypical (NT) participants who have high autistic traits (26), but can impair the differentiation between true and faked emotions (27).

People with autism spectrum disorder (ASD) typically mimic less spontaneously (28–31), which might contribute to their deficits in social cognition and social interaction. Interestingly, voluntary mimicry or inhibition of mimicry responses seems to be intact in people with ASD (30, 32). The social top–down response modulation (STORM) model proposed that rather than being impaired in mimicry per se, people with ASD, in contrast to controls, fail to modulate their mimicry according to the social context information (33). In line with this view, deficits in gaze-dependent (direct vs. averted gaze of the other) modulation of mimicry have been shown in people with ASD (34) and high autistic traits (35). The mechanism underlying such a reduced use of context is unknown, and it is not clear under which conditions spontaneous facial mimicry is reduced or intact. However, it has been suggested that attention to social input plays a major role (31). Consistent with this view, the social motivation account of ASD proposes that a diminished motivation to attend social stimuli might be causal to the social processing deficits characterizing ASD (36). This reduced social motivation might thereby result from a reduced subjective reward value of social stimuli. Evidence for this view comes from findings that infants from 6 months and young children who later develop ASD show a reduced preference for social over non-social stimuli compared to NT controls (37, 38), and similar alterations were found in adolescence (39). Further, altered brain activation in response to reward has been found in people with ASD, with some studies suggesting these alterations to be specific to (40) or stronger for social rewards (41), while others propose that monetary reward processing is affected as strongly or more strongly than social reward processing (42, 43).

Studies systematically manipulating the reward value of faces have shown that face identities paired with positive rewards are subsequently liked more and looked at longer (44) as well as remembered better (45) than those paired with negative or neural outcomes. Further, pairing faces with electrical shocks leads to conditioned responses in NT participants that are transferred within the dimension of face identity and stronger if the conditioned valence was congruent to the target face’s expression during the test phase, e.g., a negatively conditioned face with an angry expression (46). In our lab, we specifically assessed the effect of learned reward value on measures of mimicry. Participants underwent an evaluative conditioning experiment where the presence of certain face identities during a card game was associated with winning (**high reward, ‘hi’**), while other faces were associated with losing money (**low reward, ‘lo’**). When subsequently presented with the same face identities smiling at them, participants spontaneously mimicked high-reward faces more than low-reward faces as indicated by increased mu suppression (22) and facial EMG response (47). This modulating effect was negatively correlated with autistic traits (47). Using an identical paradigm on a different sample of NT participants, autistic traits were found to be negatively correlated with the reward-driven modulation of mimicry-related neural response assessed using fMRI (48). We therefore proposed that a failure to modulate mimicry levels based on reward value of a face might be a crucial piece of the puzzle in understanding atypical spontaneous facial mimicry in people with ASD, which in turn can contribute to difficulties in social bonding (47, 48).

In this study, we used the same reward-conditioning paradigm that successfully demonstrated correlated spontaneous facial mimicry (facial EMG and mu suppression) with conditioned reward values of faces (22, 47–49) to investigate whether reward value modulates mimicry-related brain activity in the IFG (50). For the first time, we compared a group of clinically diagnosed adults with ASD to a group of matched NT controls.

## Methods

### Participants

Thirty-six adults with ASD and 35 adults without any self-reported neurological or psychiatric disorder were recruited within and around the University of Reading from a database of research volunteers or advertisements. All ASD participants had a confirmed ASD diagnosis based on *Diagnostic and Statistical Manual of Mental Disorders*, *Fourth Edition* (DSM-IV) criteria from a registered clinic and were additionally assessed with the Autism Diagnostic Observation Schedule (ADOS) Module 4 (consensus of two researchers certified for reliability). All participants had normal or corrected-to-normal vision and completed a nonverbal IQ test (Raven’s Matrices). The study conforms to the norms laid out in the Declaration of Helsinki and was approved by the University Research Ethics Committee of the University of Reading, UK. All participants provided written informed consent and received either a small compensation or credit points for their participation. Ten ASD and nine NT participants were excluded, leading to a total sample of 52 participants (see **Supplementary Material** for details). Participants were matched for age, gender, handedness, and IQ between the two groups (see **Table 1**). Prior to participating in the experiment, participants (except one from the NT group) completed an online survey, including the Autism-Spectrum Quotient (51) and the Empathy Quotient (52).

**Table 1:** Sample characteristics.

Measure	NT (*n* = 26)		ASD (*n* = 26)		Statistics	*p*-value
	Mean (SE)	Range	Mean (SE)	Range		
Age	32.31 (1.90)	18–57	34.35 (2.59)	18–60	*t*-test	0.63
Gender (M:F)	17:9	−	16:10	−	Chi-square	0.77
Handedness (R:L:Amb)	21:5:0	−	19:6:1	−	Chi-square	0.45
IQ (Raven’s percentile)	46.46 (5.41)	6–90	55.96 (5.59)	2–96	*t*-test	0.23
AQ	16.48 (1.01)	6–25	37.19 (1.57)	22–49	*t*-test	<.0001

NT, neurotypical; ASD, autism spectrum disorder; SE, standard error of the mean; M, male; F, female; R, right-handed; L, left-handed; Amb, ambidextrous; AQ, autism spectrum quotient.

### Procedure

The procedure closely resembled that which was described previously in Sims et al. (48). Prior to scanning, participants underwent a conditioning phase outside the scanner where they completed an evaluative conditioning task (47). In this computerized card guessing game, a target face appeared alongside with one faceup and one facedown standard playing card, and participants guessed whether the second card would be of greater or lesser value than the first card. In the presence of one of the faces, participants won 25p to 25 pence in 90% of the trials (**hi**), while they lost 20p to 20 pence in 90% of the trials with another face (**lo**). In order to disguise the underlying structure of the game, half of the trials were paired with two further faces, where participants won and lost 60% of trials, respectively. All remaining trials were “tie” trials, i.e., the participant neither won nor lost money. Immediately after the conditioning phase, participants were positioned in a 3T Siemens Trio MRI scanner, where they completed the test phase. The test phase was designed in an event-related fashion where participants were presented with 4,000 ms video clips of the four conditioned faces making happy facial expressions. Each of the two target faces (**hi** and **lo**) was presented 30 times and each of the two additional faces 15 times. The conditioned faces were intermixed with nine unfamiliar (“oddball”) faces, and participants were asked to press a button each time an oddball face was presented in order to ensure that they were paying attention to the task. Each clip was preceded by a fixation cross, the duration of which was jittered. The stimulus order and duration of the jitter were designed to maximize power for estimating the contrast of interest, i.e., **hi** vs. **lo** (https://surfer.nmr.mgh.harvard.edu/optseq/).

During both parts of the experiment, stimuli were presented using E-Prime 2.0 (Psychology Software Tools, PA, USA). Participants took part in a different experiment reported elsewhere (53) before they were debriefed and dismissed.

### Stimuli

All stimuli were selected from the Mind Reading set (54), available at www.jkp.com/mindreading. During the conditioning phase, stimuli consisted of static images of four faces (two male and two female) with neutral facial expressions. In the test phase, stimuli consisted of four 4,000 ms video clips showing dynamic happy facial expressions made by the same four target identities. The faces were assigned to the four conditions so that they were counterbalanced between participants.

### Regions of Interest

**Figure 1:**
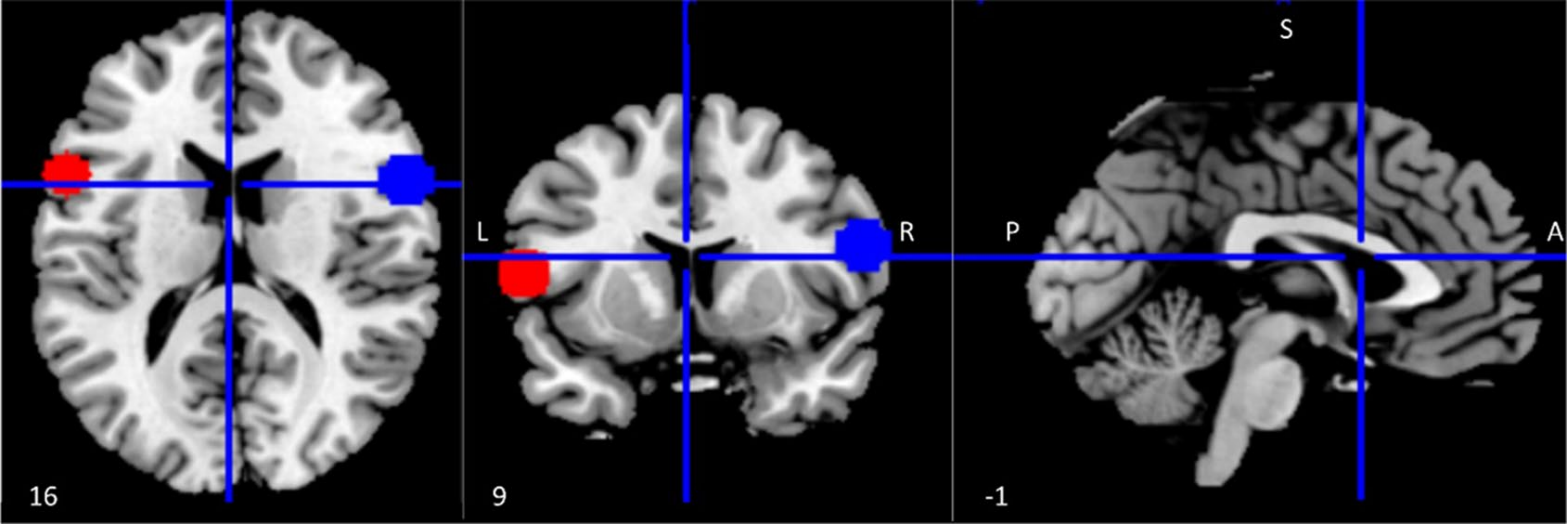
Regions of interest (ROIs) within the mirror network. ROI (10 mm radius) placement in left (red) and right (blue) inferior frontal gyrus (IFG). Coordinates were derived from a meta-analysis on action observation and imitation, corresponding to a peak of activation consistently associated during both observation and imitation of facial expressions as well as movements of other body parts (15).

Regions of interest (ROIs) within the left and right inferior frontal gyrus (IFG) were identified using coordinates reported in a published meta-analysis of 139 neuroimaging studies of action observation and imitation as peak activations from the conjunction analysis between observation and imitation of face, hand, finger, leg, and foot movements in humans (15). The Wake Forest University (WFU) Pickatlas tool (55) was used to draw spheres with a 10 mm radius around the center coordinates [left IFG (LIFG) = (−56 12 10); right IFG (RIFG) = (58 15 10)] of the selected ROIs (**Figure 1**).

### FMRI Data Acquisition and Preprocessing

Participants were scanned in a 3T Siemens TIM Trio MRI scanner with a 32-channel head coil. 32 slices, 3-mm-thick axial slices were acquired in descending sequential order. A multi-echo sequence with three different echo times [Repetition time (TR) = 2,400 ms; Echo time (TE) (1; 2; 3) = 20; 36; 52 ms] was used in order to increase the signal-to-noise ratio (56–58). Preprocessing and multi-echo independent component analysis (ICA) (58) were performed in analysis of functional neuroImages (AFNI) (59). The first four volumes were discarded to allow for the stabilization of the magnetization. The data were further preprocessed using slice-timing correction, motion correction, and the functional-to-structural coregistration. Subsequently, the multi-echo ICA was performed in order to enable separating blood oxygen level dependent (BOLD) from non-BOLD components. The non-BOLD components were used as nuisance regressors to denoise the functional data, which were then converted to 3-D images with dcm2nii, normalized to Montreal Neurological Institute (MNI) space, and spatially smoothed with a Gaussian kernel of full width at half maximum (FWHM) 5 mm using statistical parametric mapping version 8 revision 6313 (SPM8) (www.fil.ion.ucl.ac.uk/spm).

### FMRI Data Analysis

Statistical parametric maps were calculated using SPM8 with multiple regressions of the data onto a model of the hemodynamic response (60). The first-level general linear model analyses contained five regressors of 4,000 ms duration for the five conditions, i.e., **hi**, **lo**, the two distractor faces (60% win and loss, respectively), and the oddball faces. Regressors were convolved with the canonical hemodynamic response function. Mean *t*-statistics of the contrast [**hi > lo** faces] for each participant was extracted for the left and right IFG ROIs with MarsBaR (version 0.44) and used as dependent variables for the group-level analysis.

To test both categorical as well as dimensional approaches, two models of ordinary least-squares regression were computed. The first model tested the effect of group, while the second model tested the effect of autistic traits autism spectrum quotient (AQ). Mean ± 3SD was used as the criterion to filter outliers, and none were identified. Due to the directional nature of the hypothesis (i.e., IFG activation for **hi** vs. **lo** faces would be reduced in participants with ASD as compared to controls), one-tailed *p*-values are reported.

## Results

### Behavioral Outcomes

We evaluated the task performance during the test phase to verify that participants attended to the task. None of participants had more than two misses in the test phase, indicating that they attended the stimuli. Two participants from the ASD group and two from the control group had more than two false alarms. These participants were included in the analyses reported below. To guard against the possibility of the data from these participants having an undue influence on the reported results, all analyses were rerun after excluding them, which confirmed that the results remained the same (see **Supplementary Material**). For the remaining participants, the low number of false alarms (accuracy above 97%) indicates that they recognized the conditioned faces.

### fMRI Results

**Figure 2:**
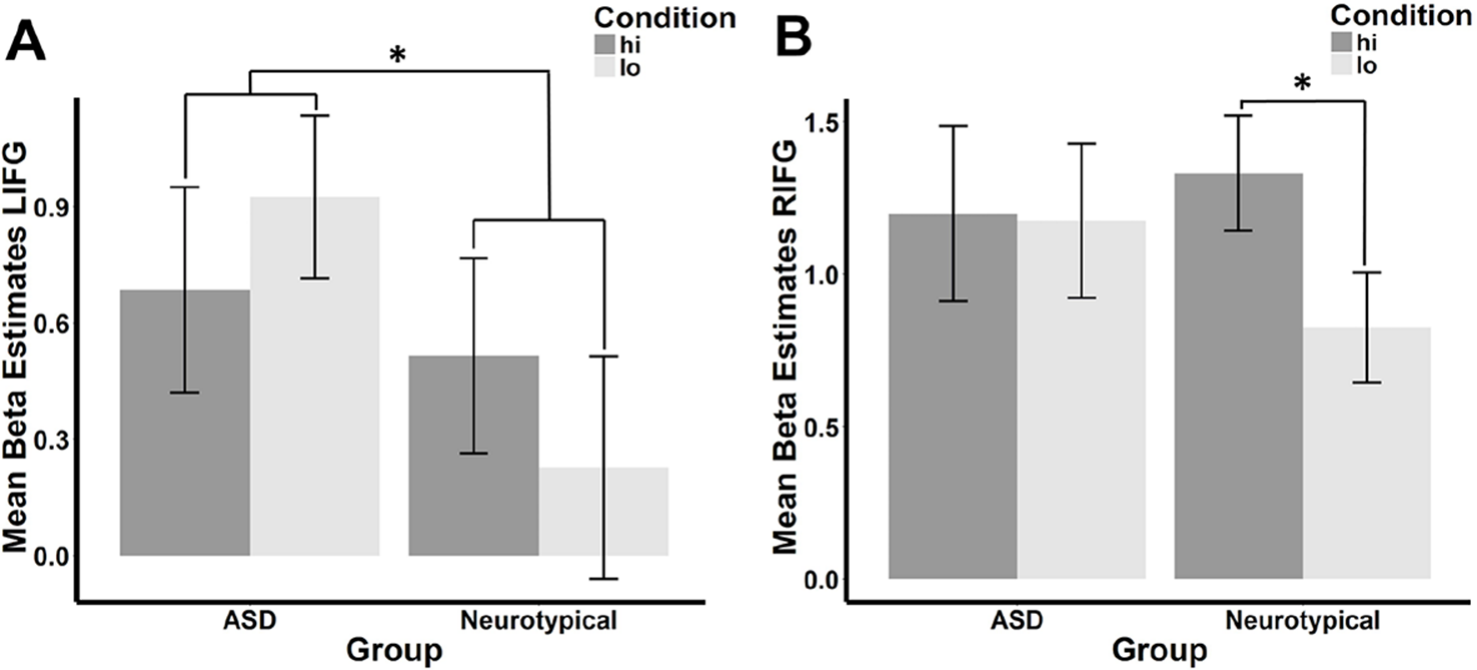
Mean beta estimates per group (ASD vs. neurotypical) and condition in the **(A)** left and **(B)** right IFG. Mean beta estimates in response to faces conditioned with high reward (**hi**) are marked in dark gray, while those in response to faces associated with low reward (**lo**) are marked in light gray. Asterisks indicate significant differences, a group-by-condition interaction in the LIFG, and an effect of condition in the neurotypical group in the RIFG.

There was a significant interaction between group and condition within the LIFG (**β** = −.287, *p* = .033), but this interaction fell below the standard threshold of significance for RIFG (**β** = −.250, *p* = .069). Planned *post hoc* analyses revealed that the direction of the **hi>lo** contrast was inverse between the ASD and NT groups in the LIFG (see **Figure 2A**). When each group was considered separately, the difference between conditions was not significant (ASD: *t* = −1.193, df = 25, *p* = .878; NT: *t* = 1.312, df = 25, *p* = .101). In the RIFG (see **Figure 2B**), neural response was significantly stronger for **hi** compared to **lo** faces in NT controls (*t* = 2.092, df = 25, *p* = .023), while no significant difference was observed in the ASD group (*t* = .112, df = 25, *p* = .456).

**Figure 3:**
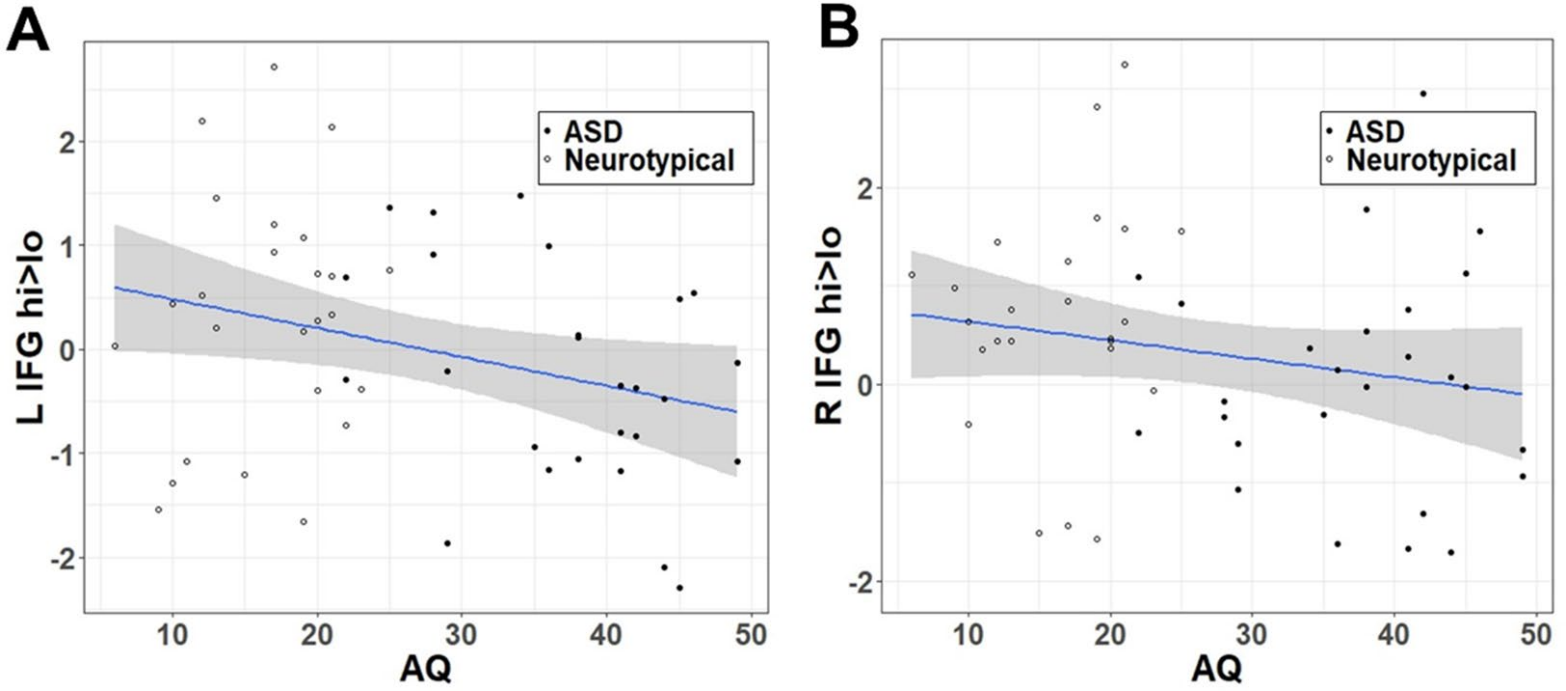
Correlation between **hi>lo** beta contrast within the IFG ROI and autistic traits (AQ) in **(A)** left and **(B)** right hemisphere.

Similar to the effect of group within the categorical model, autistic traits (**Figure 3**) were negatively correlated with the **hi>lo** contrast within the LIFG (*r* = −.303, *t* = −2.224, df = 49, *p* = .0154) but fell short of the *p* < 0.05 threshold in the RIFG (*r* = −.198, *t* = −1.414, df = 49, *p* = .082).

There was no difference between the NT and ASD groups in head motion as indicated by the euclidean distance between head position parameters [mean(TD) = 0.078; mean(ASD) = 0.083; *t* = −0.435, df = 48.466, *p*(2-sided) = 0.665].

## Discussion

Spontaneous facial mimicry is atypical in people with ASD, but the underlying mechanism is unclear. By using an evaluative conditioning paradigm for associating different reward values with different faces, we found that reward value modulated mimicry-related brain activity in the IFG (50) differentially in individuals with ASD vs. NTs. Specifically, NT individuals showed greater IFG response to faces that were associated with high vs. low reward than those with ASD. Additionally, NT but not ASD participants had significantly more RIFG activation for the **hi** faces as compared to the **lo** faces. The results support the notion that the effect of learned reward value on spontaneous facial mimicry is altered in ASD.

### Reward Modulates Mimicry-Related Brain Response in Neurotypicals

The stronger activation of the IFG as a core component of the human mirror system (15) when passively viewing smiling faces associated with high vs. low reward value observed in NT participants is in line with previous findings that more rewarding faces are mimicked more (47). It also corresponds to findings indicating that humans typically mimic whom they like better more (12–14). Similarly, we found in a previous study that NTs had increased functional connectivity between the IFG and the right ventral striatum, a core region for reward processing, for high- vs. low-reward faces (48).

### Atypical Reward-Dependent Modulation of Mimicry-Related Brain Response in Autism Spectrum Disorder

In our ASD participants, there was no increased IFG response to faces associated with high vs. low reward in either hemisphere, suggesting that reward conditioning did not modulate mimicry-related brain activity in the same way in these individuals. It is unlikely that the lack of a difference between high- and low-reward faces in IFG activation results from a failure to learn to associate the conditioned reward value with the faces in ASD participants. First, participants from both groups rated the high-reward faces as more likeable than the low-reward faces after conditioning, and this effect showed no interaction with group (see **Supplementary Material**). Second, one of our previous studies demonstrated that people learn to implicitly associate reward with faces, irrespective of their autistic traits (49). In accordance with these previous results and the hypothesis of a weaker link between reward and mimicry in ASD (44, 45, 48), we propose that rather than failing to learn to adjust the reward value of faces in the current study, participants with ASD did not use the learned reward value for adjusting their spontaneous facial mimicry to the same extent as NT participants did. Similarly, it has been proposed that people with ASD make less use of social context information (such as direct vs. averted gaze) than controls in order to modulate their mimicry (33). Hence, rather than being impaired in mimicry per se, people with ASD might typically modulate their mimicry response less, due to atypical reward-driven modulation. This observation can provide a parsimonious explanation for the atypical contextual modulation of mimicry effects reported in ASD. It might also explain why some studies report reduced spontaneous mimicry (28–31) in ASD, while studies of deliberate mimicry or those involving inhibition of a preplanned movement (often used as a marker of automatic mimicry) do not observe group differences (30, 32, 61–63). Previous observations of altered social and nonsocial reward processing in ASD (40, 42, 64–67) lead to the question of whether these atypical reward-modulation effects detected here are specific for social stimuli. The current study does not answer this question, since there is no control condition with nonsocial stimuli. However, in a previous study, we have shown that the impact of autistic traits on reward modulation of automatic mimicry was seen for social but not nonsocial stimuli (68). Future studies should explicitly test this possibility using the current paradigm.

In studying adults cross-sectionally, it remains unclear whether the deficient reward–mimicry link is a cause or consequence of altered trajectory in brain development. Future studies should investigate this link longitudinally in young children. Additionally, since only high-functioning ASD individuals were included in the current study, our results do not necessarily generalize to the entire autism spectrum. Future studies should test this question in the more severe end of the spectrum (69). Additionally, the variability of mean beta estimates was higher in our ASD than in our NT sample, as indicated by higher standard errors. This suggests that the modulating effect of reward on mimicry might not be reduced in all ASD participants and correspond to previous findings of more variability in behavioral and neural response outcomes in ASD vs. NT samples (70).

### Categorical vs. Dimensional Accounts of Autism Spectrum Disorder

To account for the view of ASD as the extreme end of a spectrum of continuously distributed traits (71), we further conducted dimensional analyses where we tested the relationship between the IFG response to high- vs. low-reward faces and autistic traits. Similar to the categorical account, we observed a negative association between autistic traits and the high- vs. low-reward contrast within the LIFG. This result corresponds to previous findings from our group in a different sample, indicating that autistic traits predict the extent of reward-driven modulation of spontaneous facial mimicry (47, 48). Together with these previous findings, the results therefore confirm a similar effect of both categorical and dimensional accounts of ASD on the modulating effect of reward on mimicry. While this relationship was significant across the whole sample, it was driven by the greater range of AQ scores in the ASD group.

### A Bidirectional, Weakened Link Between Mimicry and Reward in Autism

Just as reward modulates mimicry, being mimicked by others is also perceived as rewarding (72). NT females show an immediate reward-related brain response while observing others being mimicked (73), and emotional synchrony between primed and presented emotion leads to reward response regardless of the emotional valence (74). We have previously demonstrated that faces that consistently mimic the participant are liked more and looked at longer compared to faces that consistently perform the facial expression opposite to that of the participant (44). In another study in our lab, we used the same mimicry conditioning to demonstrate that mimicking as compared to anti-mimicking faces evoked stronger reward-related activation of the ventral striatum in NT but not ASD participants (53). Together with these previous findings, the current study contributes to the evidence for a weakened link between reward and mimicry people with in ASD in both directions. Given the potential role of mimicry in both social cognition (*via* embodied cognition) and building rapport, a weakened bidirectional link between reward and mimicry could be a key mechanism underlying difficulties in social interaction in ASD.

## Ethics Statement

The study conforms to the norms laid out in the Declaration of Helsinki and was approved by the University Research Ethics Committee of the University of Reading, UK. All participants provided informed consent.

## Author Contributions

BC developed the study concept. BC and JN contributed to the study design. Data collection was done by JN. C-TH and JN contributed to the analysis. The draft of manuscript was written by JN, while both other authors contributed to critical editing of the draft. All authors approved the final version of the manuscript for submission.

## Funding

This work was supported by grants to BC from the Medical Research Council UK (G110359/1) and Leverhulme Trust (PLP-2015-329).

## Conflict of Interest Statement

The authors declare that the research was conducted in the absence of any commercial or financial relationships that could be construed as a potential conflict of interest.
