# Supplementary material for: Atypical Reward-Driven Modulation of Mimicry-Related Neural Activity in Autism
Source: Front Psychiatry. 2019 May 16;10:327. doi: 10.3389/fpsyt.2019.00327 (PMC6532344; doi:10.3389/fpsyt.2019.00327)
Supplement: Supplementary file 1 [file DataSheet_1.docx]

Supplementary Material

Atypical Reward-driven Modulation of Mimicry-related Neural Activity in Autism

Neufeld J, Hsu C-T, Chakrabarti B*

*** Correspondence:** Corresponding Author: b.chakrabarti@reading.ac.uk

# Supplementary Data

Participant exclusion

Exclusion reasons were current usage of anti-psychotics (one ASD participant), unverified ASD diagnosis (one ASD participant), being under-aged (one ASD participant), the inability to finish the experiment (three ASD participants), misunderstanding the test phase task (one NT participant), scanner failure (one NT participant), structural anomaly (one NT participant), data loss (one ASD participant), excessive head movements (one ASD and three NT participants) and other fMRI data quality issues (two ASD and three NT participants).

Verification analyses

Out of the twenty-six included ASD participants, six of which four were females had ADOS total scores below the ASD-cutoff. In order to exclude the possibility that these individuals might have been driving the effect, we re-run the analysis after excluding these ASD participants. The results remained largely the same with significant effects remaining (LIFG: beta (ASD-NT) = -.736, p = .019; RIFG: beta (ASD-NT) = -.499, p = .092).

Similarly, we also repeated the analyses after excluding two participants from the ASD group and two from the NT group who had more than two false alarms during the test phase, leading to similar results. The group*condition interaction was still significant in the LIFG (β=-.583, p=.038), but not in the RIFG (β=-.454, p=.102). AQ scores remained negatively correlated with the **hi>lo** contrast within the LIFG (r=-.283, p=.027), but not in the RIFG (r=-.173, p=.123).

Face rating results

Before the conditioning and after completion of the test phase participants rated all face stimuli on a seven-point Likert scale for likeability (1 = not likeable at all, 7 = very likeable) and attractiveness (1 = not attractive at all, 7 = very attractive). The likeability and attractiveness ratings for **hi** vs **lo** faces before and after conditioning were analysed using repeated measures ANOVAs with condition (**hi vs lo**) and conditioning (before vs after conditioning phase) as within-subject factors, and ASD diagnosis as a between-subject factor. This was done in order to confirm an explicit change in the reward value of the face after conditioning. Rating data from one NT and one ASD participant were lost due to technical errors. For likeability rating, there was a significant interaction between condition and conditioning (F=4.710, p=.030), indicating an increase in likeability due to the conditioning for hi faces only. These results indicate that the conditioned reward value was learned and translated into liking in both controls and participants with ASD alike. For attractiveness, only the main effect of conditioning was significant (F=5.692, p=.018) with higher ratings after conditioning, indicating that subjectivity perceived attractiveness increased with familiarity for all faces so irrespective of the learned reward value. There were no significant main effects of group nor three-way interactions between group, condition and conditioning for either rating modality.
